# Post-Treatment Adverse Health Correlates among Prostate Cancer Survivors in a Sample of Men Residing in Atlantic Canada

**DOI:** 10.3390/curroncol28040246

**Published:** 2021-07-25

**Authors:** Gabriela Ilie, Robert Rutledge, Ellen Sweeney

**Affiliations:** 1 Department of Community Health and Epidemiology, Dalhousie University, Halifax, NS B3H 4R2, Canada; 2 Department of Urology, Dalhousie University, Halifax, NS B3H 4R2, Canada; 3 Department of Radiation Oncology, Dalhousie University, Halifax, NS B3H 4R2, Canada; Rob.Rutledge@nshealth.ca; 4 Atlantic PATH, Dalhousie University, Halifax, NS B3H 4R2, Canada; Ellen.Sweeney@Dal.Ca

**Keywords:** depression, anxiety, prostate cancer, cancer, mental health, survivorship, quality of life, psychosocial oncology

## Abstract

Background: Recent large population-based studies have shed light on an association between prostate cancer (PCa) survivorship and mental health, which emerged when the comparison group was either men without a history of cancer or those with any other type of cancer except prostate. Here we examine the role of surgery alone, compared to other types of treatment modalities in this association in a population-based sample of men with prostate or other types of cancer. Methods: A cross-sectional analysis was conducted on a subsample of 632 male participants aged 36–69 from the 2009–2015 survey cycle of the Atlantic PATH cohort study. The primary outcomes were the presence of mild, moderate or severe depression or anxiety indicators and were assessed using the seven-item generalized anxiety disorder (GAD-7) scale and the nine-item Patient Health Questionnaire (PHQ-9), respectively. The presence of a lifetime history of PCa or other form of cancer (except PCa) was the main predictor variable and was assessed in cancer treatment modality (surgery or other types of treatment modalities) stratified analyses. Covariates included age, marital status, household income, comorbidity, and survivorship time. Results: The presence of depression in this sample was prevalent among 17.7% of men, and of anxiety among 9.3% of men. Survivors who were treated with surgery for their PCa diagnosis had 7.55 statistically significantly higher odds of screening positive for current depression symptoms compared with those of other forms of cancer in controlled analyses. These differences were not observed for anxiety. Conclusions: These findings emphasize the need for multidisciplinary survivorship care plans among PCa patients, especially those who undergo surgery. Targeted programming aimed at prioritizing and delivering comprehensive mental health support to PCa survivors early in the survivorship journey is justified.

## 1. Introduction

Prostate cancer is the second most common cancer in men worldwide and the fifth leading cause of men’s cancer death [1]. However, prostate cancer carries one of the most favorable five- and ten-year survival rates of all malignancies [2]. With over 1.1 million men diagnosed annually, there is an ever growing population of patients living with a prostate cancer diagnosis [3,4,5,6]. As such, it is becoming increasingly important to understand what unmet survivorship needs may lead to poor quality of life and short- and long-term mental health issues in this population [7,8,9,10,11,12]. Prostate cancer treatments including surgery, radiation, hormone therapy, chemotherapy, or a combination are highly effective, but are also plagued by a host of life-altering side effects. Physical side effects may include urinary, bowel and sexual dysfunction [13,14,15]. In recent years, mental health issues emerging from unmet survivorship needs among prostate cancer survivors revealed a silent epidemic of loneliness and disconnect [7,9,12]. A recent report presents evidence of an association between poor mental health and psychological morbidity and biopsychosocial variables, including poor oncological outcomes, particularly but not limited to advanced forms of the disease [8]. More than one in two men diagnosed with prostate cancer experience mental health distress [16], with 10–40% scoring positive for clinically significant depression at various points during their survivorship journey [9,12,17,18]. Younger age, Caucasian ethnicity, urinary problems and erectile dysfunction, dissatisfaction in relationships with current partner, substance abuse, and multimorbidity have been identified as factors predicting depression and/or anxiety in prostate cancer survivors [9,10,11,12]. When compared to the general population, cancer patients are disproportionately affected by suicidality (31.4 suicides per 100,000), with prostate cancer patients among the most at-risk for death by suicide (48.3 suicides per 100,000) [4,19,20]. The most salient risk factors for suicide attempts among prostate cancer survivors include Caucasian ethnicity, older age, male gender, living alone, and distant disease as men are most at risk for suicide >15 years after diagnosis [4]. 

Furthermore, recent evidence from large population-based studies demonstrated that men diagnosed with prostate cancer have more than double the odds of mental health issues compared with men with no history of cancer and men with any other type of cancer except prostate [9,12,17]. Few studies, however, have been conducted examining the relationship between treatment modality among males with a history of cancer, cancer type, and the potential association with mental health illness in this population [11]. However, the identification of adverse health correlates with poor mental health among male cancer survivors is critical for the development of survivorship care plans of survivors of prostate cancer. Here we evaluate the contribution of treatment modality to the association between men’s cancer type (prostate or other forms of cancer) with depression and anxiety during cancer survivorship in analyses controlled for survivorship time, age, marital status, household income, and multimorbidity. 

## 2. Methods

### 2.1. Procedure

This study was based on a subsample of 632 men (ages 35 to 69 years old, M = 59.37 years old) who completed a health questionnaire between 2009 and 2015 as part of baseline data collection in the Atlantic Partnership for Tomorrow’s Health (Atlantic PATH) cohort, and who had a history of a cancer diagnosis. Atlantic PATH is a regional cohort in the Canadian Partnership for Tomorrow’s Health (CanPath, formerly the Canadian Partnership for Tomorrow Project), a pan-Canadian longitudinal cohort study investigating the role of genetic, environmental, behavioral, and lifestyle factors in the development of cancer and chronic disease [21]. The men in this study were residents of one of the four Atlantic Canada provinces (Nova Scotia, New Brunswick, Prince Edward Island, and Newfoundland and Labrador). This study was reviewed and found to be in accordance with the ethical standards of Dalhousie University. Survey procedures were in accordance with the ethical standards of the responsible committee on human experimentation and with the Helsinki Declaration of 1975, as revised in 2000. All participants provided written informed consent. Details on recruitment and data collection have been previously described [22,23]. Participants were recruited through advertising, media coverage, community and workplace events, incentive programmes (e.g., Airmiles) and community champions who encouraged their friends and families to participate. Participants completed a set of standardized surveys on sociodemographic characteristics, health history and lifestyle factors, as well as providing physical measures and biological samples. 

### 2.2. Measures

*Mental Health–Depression* was one of the two primary mental health outcome measures and was assessed using the Patient Health Questionnaire (PHQ-9). This self-reported questionnaire assessed depressive behaviors over the past two weeks through nine questions based on DSM-V criteria that screen for the presence or absence of clinical depression [24,25,26]. Responses range from “not at all,” “several days,” “more than half the days,” and “nearly every day,” coded 0 to 3, respectively. Scores below 5 indicate good mental health, while scores of 5–9, 10–14, 15–19, and 20–27 indicate mild, moderate, moderately severe, or severe depression, respectively. To ensure adequate cases, scores on PHQ-9 were binary coded to depict the absence (below 5, coded 0) or presence (above 5, coded 1) of clinical depression. The reliability coefficient Cronbach’s alpha for the PHQ-9 is 0.79 among men [27]. The Cronbach’s alpha obtained in our study for the PHQ-9 was 0.72. 

*Anxiety* was the second mental health outcome measure and was assessed using the generalized anxiety disorder scale (GAD-7) [28]. Participants were asked to report anxiety symptoms experienced over the past two weeks using seven questions evaluated using four responses of zero (“not at all”), one (“several days”), two (“more than half the days”), or three (“nearly every day”). Scores on GAD-7 range between 0 and 21, with scores under 5 indicating good mental health (coded 0) and 5–9 indicating mild, 10–14 indicating moderate, and 15–21 indicating severe clinical anxiety (coded 1). The binary coding was chosen to ensure adequate cases. The dichotomization of the presence or absence of mental health symptoms of mild or greater severity is common in the literature [29]. The internal consistency of the GAD-7 is very good (Cronbach’s α = 0.91) and the associations of GAD-7 sum scores with self-report measures of depression and social anxiety supported construct validity [30]. The Cronbach’s alpha obtained in our study for the GAD-7 was 0.84. 

### 2.3. Predictors

*History of cancer diagnosis.* Participants were asked to identify if they have ever been diagnosed with cancer, their age at diagnosis and type of cancer. Type of cancer included bladder (*n* = 23), brain (*n* = 4), breast (*n* = 5), colon (*n* = 56), esophagus (*n* = 8), kidney (*n* = 21), larynx (*n* = 4), leukemia (9), liver (3), lung and bronchus (*n* = 10), non-Hodgkin’s lymphoma (*n* = 35), prostate (*n* = 140), rectum (*n* = 8), skin (*n* = 228), stomach (*n* = 4), thyroid (*n* = 16), and other (*n* = 58). The presence of a lifetime history of PCa diagnosis was coded 1, the presence of a lifetime history of other type of cancer was coded 0. 

*Treatment modality* was the main predictor. To ensure adequate cancer cases, two types of treatment modalities were contrasted: surgery exclusively (*n* = 343, coded 1) versus other types of treatment (radiation, *n* = 70, chemotherapy, *n* = 111, and other or combined types of treatment, *n* = 108, coded 0).

### 2.4. Covariates 

Five covariates were included in the model and included age, relationship status, household income, survivorship time, and comorbidities. For relationship status, participants married or living with partner were coded as 1, and those divorced, widowed, separated, single, or never married were coded as 0. Household income was coded as 1 for under $50,000 CAD, 2 for $50,000–99,999 CAD, and 3 for $100,000 CAD or more. To ensure that our findings were not influenced by any changes in sample attributes across months of survivorship, we included survivorship time (months elapsed between the first cancer diagnosis and survey completion) as a covariate. Lastly, absence of additional comorbidities was coded 0, and the presence of one or two or more comorbidities was coded 1 and 2, respectively. 

### 2.5. Statistical Analysis

A power analysis using G*Power 3.1. software was performed to evaluate the adequacy of the sample size for the analyses undertaken. All analyses were performed with SPSS V26. Cross-tabulation analyses were used to first assess the association between screening positive for depression and anxiety and the stated predictors and covariates. A missing variables analysis revealed that 54% of the anxiety and 52.7% of depression variable data were missing. Little’s MCAR test was statistically significant (*p* < 0.001), indicating that data were not missing at random. Although a visual examination of the missing data did not reveal any systematic patterns, multiple imputation (MI) was used to supplement the analyses and add confidence to the results obtained. MI was performed using SPSS V.26, using an iterative Markov chain Monte Carlo (MCMC) algorithm known as fully conditional specification (FCS) or chained equations imputation. The number of imputations, 73, was randomly generated to represent a value within the range of 33 to 100, as recommended by the literature [31,32]. Two multivariate logistic regression analyses assessed the association between the stated predictors and covariates and the presence or absence of a positive screening test for clinical depression or anxiety. Prior to conducting the analyses, the assumptions of logistic regression were tenable. Reported analyses include the pooled MI results to assess comparison tenability. After listwise, the analytical sample was 268. 

### 2.6. Sensitivity Analyses

To determine if the results obtained were contingent on how the missing data were handled, sensitivity analyses were conducted. Results were pooled in a summary measure following MI analyses with analytic sample of 268 for each of our two outcomes (depression and anxiety) and were compared with the listwise exclusion original dataset which had analytic samples of 299 and 291 responses for each of our two outcomes (depression and anxiety), respectively.

## 3. Results

In this population-based sample, 22.1% of men reported having a history of PCa diagnosis and 77.9% reported having a history of other forms of cancer. An estimated 17.7% (*n* = 53) and 9.3% (*n* = 27) of men in the total sample screened positive for the presence of depression or anxiety, respectively.

Table 1 presents descriptive and separate logistic regression analyses for each of the two outcomes as they relate to each predictor and covariate. Most men in the sample were surveyed a year post their diagnosis, received surgery exclusively for their cancer diagnosis, were married, reported a household income of $50,000 CAD and above, and had multiple comorbidities at the time they took the survey.

Table 2 presents treatment stratified multiple logistic regression analyses predicting the presence or absence of depression or anxiety. The fitted model for surgery was statistically significant (X^2^(8) = 32.91, *p* < 0.001) and stable (Hosmer and Lameshow X^2^(8) = 4.24, *p* > 0.05), predicting 41% of depression (Nagelkerke R^2^ = 0.41). Results of this analysis revealed that men with a history of prostate cancer who were treated with surgery for their cancer diagnosis had 7.55 (OR_MI_ = 5.09) statistically significant higher odds for screening positive for current depression status, compared to those with other types of cancer who were treated with surgery for their cancer diagnosis. Men who identified as being divorced, widowed, separated, or single/never married had statistically higher odds, OR = 16.48 (OR_MI_ = 6.18) for screening positive for depression compared with men who were married or currently in a relationship. Household income was a marginally significant contributor to depression in the fitted model, while comorbidity and survivorship time since diagnosis did not statistically significantly contribute to differentiating between the presence or absence of depression in the surgery treatment analysis. The fitted model for surgery was not statistically significant for predicting anxiety (X^2^(8) = 14.08, *p* > 0.05), although stable (Hosmer and Lameshow X^2^(8) = 9.58, *p* > 0.05). Men with a history of PCa cancer who were treated with surgery for their cancer diagnosis had comparable anxiety levels with those with a history of other forms of cancer treated with the same treatment modality. Except for household income, with an OR = 12.06 (OR_MI_ = 10.04, ns), none of the covariates statistically significantly contributed to the presence or absence of anxiety in this subsample.

The multiple logistic regression fitted model for the other forms of cancer treatment modalities was not statistically significant (*p* > 0.05) for depression, but indicated a fit (X^2^(8) = 26.52, *p* < 0.01, Nagelkerke’s R^2^ = 0.41) and a stable model for anxiety (Hosmer and Lemeshow Test X^2^(8) = 0.85), with younger ages (36 to 59) (OR = 16.31, OR_MI_ = 13.42) and one (OR = 8.69, OR_MI_ = 5.82) or more (OR = 30.63, OR_MI_ = 57.89) comorbidities in addition to the diagnosis of cancer contributing to the presence of anxiety among men with a history of cancer diagnosis.

## 4. Discussion 

Cancer survivors experience the physical, psychosocial and emotional effects of the disease and its treatment, which vary between individuals, cancer type, stage of diagnosis, treatment, and survivorship time. Physical side effects, as well as cognitive limitations, coping issues, fatigue, depression, and anxiety impact cancer survivors’ overall quality of life, relationship satisfaction, ability to return to work, and long-term survival [12,33]. Many cancer survivors experience clinically significant levels of anxiety and depressive symptoms and reduced overall mental wellbeing throughout the cancer journey, including after treatment [33]. A national study conducted by the Canadian Partnership Against Cancer found that “most cancer survivors experience mental health challenges upon completion of treatment, and many do not receive treatment and care as they transition back to their daily lives [34,35].” 

A systematic review found that prostate cancer survivors reported the following individual needs in order of frequency: health system/information needs; interpersonal/intimacy needs; psychological/emotional needs; physical needs; family-related needs; patient-clinician needs; daily living needs; practical needs; spiritual needs; and social needs [36,37,38]. It identified a wide range of unmet supportive care needs, particularly in the areas of intimacy, informational, physical and psychological needs [38]. While there are Canadian guidelines for prostate cancer screening practices from organizations such as the Canadian Task Force on Preventive Health Care and the Canadian Urological Association [37,39], a comprehensive approach to short- and long-term survivorship care is often lacking. It is important that patients are fully informed about the potential side effects of treatment, including surgery, and that mental health support is provided in the short- and long-term. New studies have emerged in the past few years stressing the importance of providing multidisciplinary education and survivorship care to survivors of prostate cancer, given the higher rates of depression and anxiety compared to men with any other form of cancer in the short and long term [7,8,9,10,11,12]. 

This study population is demographically consistent with other population studies that have investigated the relationship between the presence of a history of a cancer diagnosis and mental health [7,8,9,10,11,12]. It contributes to the body of literature in its findings that prostate cancer survivors cannot be broadly categorized with the mental health needs of other cancer survivors and, furthermore, there are differences among prostate cancer survivors themselves. Recent population-based studies have determined that men with a history of prostate cancer in Canada and Atlantic Canada had 1.24 and 2.05 higher odds of depressive symptoms, respectively, compared to men without a lifetime history of prostate cancer [9,12]. Prostate cancer survivors were identified as having 10.23 times higher odds of anxiety symptoms than other cancer survivors [9,12]. While these findings are not consistent with smaller (21 patients) scale studies on the mental health of prostate cancer survivors and type of treatment [40], in this case, prostate cancer survivors who were treated with surgery had 7.55 statistically significantly higher odds of screening positive for current depression symptoms when compared to survivors of other types of cancer who were treated with surgery.

Unlike other forms of cancer (our sample included skin, colon, kidney, breast and thyroid, where surgery would have caused fewer long-term physical, functional and psychosocial side effects), prostate cancer surgery can result in urinary problems, erectile dysfunction, and changes in sexual behavior and intimacy—issues that may be identified by patients as major concerns, in the short and long term [10,11,14,15,41]. Given that most men affected by prostate cancer are between 50 and 65 years of age (younger), have earlier disease (due to increases in early detection), and are fitter (e.g., able to undergo major surgery), these side effects can take a heavy toll on the psychological and social well-being of these men, especially younger men [10,11]. Another important consideration is that surgery is usually given to cancer patients who have early, highly curable cancer [3]. As such, the threat of cancer recurrence is less of a worry for the surgery-only survivors (prostate and others) compared to the comparison group (other active forms of treatment). This was reflected in the lack of anxiety illness among surgery patients. Furthermore, the high number of skin cancers (*n* = 228) in the “other cancers” group also points to a large cohort of well patients (no detectable presence of disease) at the point of taking the survey. Therefore, it is likely that the significant increased odds (seven times higher) among prostate cancer survivors to screen positive for clinical depression (but not anxiety), compared to other types of cancer comparison group, could be due to the psychosocial side effects (urinary incontinence, sexual dysfunction, social isolation, loneliness, disconnect) of surgery following prostate cancer, which are picked up later after the treatment has been completed [3,10,11]. Other factors that contributed significantly to the depression predictive model, in the surgery group, were being single, divorced or widowed, and low household income in the past 12 months. Taken together, when these biopsychosocial factors emerge, it is not surprising that clinical depression is noted among the prostate cancer survivors group. These findings combined with other emerging results in the literature point to the need for clinicians to be vigilant to screen for depression in prostate cancer patients who show poor social determinants of health, especially when surgery is chosen as the choice of treatment for their disease [8,17].

Retrospective survey data for cancer patients, three to six months post-care, suggest that patients may not always receive sufficient information on the physical side effects of their surgery, which include the emotional and sexual changes, or the effects these side effects may have on their relationships and quality of life [36]. The most negative rating in the survey was the emotional support received. While levels of anxiety are often highest at the time of prostate cancer diagnosis [36], results we report here and those reported by recent research demonstrate that depression among prostate cancer survivors occurs throughout the cancer journey [7,8,9,10,11,12]. 

Researchers have previously discussed the impact of prostate cancer surgery side effects on patients’ social and psychological impact. Hanly et al. (2014) identified three inter-connected themes related to prostate cancer survivors’ mental health and psychosexual adjustment, including (i) psychosocial impact, (ii) communication and support, and (iii) integration process on prostate cancer patients’ psychosocial adjustment [42]. Prostate cancer patients are reported to experience distressing sexual and urinary difficulties, as well as altered self-perception and intimate relationships [10,11,42]. Improved communication with doctors and partners as well as the provision of comprehensive information and support are identified as key factors in facilitating coping and improved quality of life post treatment [42]. These findings are also consistent with others that highlight the need for integrated emotional and mental health support from the care team throughout the cancer journey, particularly after treatment [36,43]. There are limited guidelines that address the provision of follow-up care for prostate cancer patients after treatment [41]. Patients would benefit from survivorship care plans that adequately encompass and address their unique needs. A multidisciplinary approach including all members of a health care team is recommended to support long-term physical and mental wellbeing through health promotion, surveillance for recurrence, screening for secondary cancers, long-term and late effects assessment and management, psychosocial issues, and care coordination [24,41,42].

Cancer surveillance programs such as the Atlantic PATH are critical aspects of survivorship and can be spearheaded by cancer care providers or research groups. The American society of Clinical Oncology has provided recommendations for prostate cancer education and survivorship care providers in 2014 to help guide survivorship care across various provider settings [44]. Within these guidelines it is recommended that depression and anxiety be screened for, as part of ongoing psychosocial assessments during oncological check in assessments at 6 months, 12 months or annually, depending on the checking points. Screening tools such as PHQ-9, the Expanded Prostate Cancer Index Composite for Clinical Practice (EPIC-CP) and a suicidal ideation questionnaire may be used throughout the cancer journey, including after treatment, to identify patients who may be at high risk [4]. Patients should also be informed about a range of support resources including counselling, educational seminars and support groups [6,14,43,45]. It has been suggested before that a multidisciplinary approach can lead to improved delivery of care in prostate cancer [44]. Employment of a survivorship model that includes mental health assessment is good for the patient, it encourages collaboration across several specialties and improves routine health maintenance. 

### Limitations

While these analyses had enough power to detect if an effect was present, there were not enough cell counts present to analyze specific types of cancer other than prostate due to a large percentage of missing data in the outcome variables. Future studies should attempt to replicate these results with other types of cancer and larger sample sizes. The cross-sectional, retrospective and self-reported nature of the study is a limitation, and causal inferences cannot be made. Given that the relationship status variable in this study was reduced to two categories to avoid small cell counts (<5), future studies of larger sample sizes should include stratified analyses that may allow for the examination of the role of various relationship statuses as possible at-risk factors. The data reported here may have also been subject to recall bias. Survival bias may be a potential concern. Future studies of a larger sample size using mixed-method design may consider evaluating the contribution of other variables, such as ethnicity, the presence or absence of cancer recurrence, and the interaction with other cancer diagnoses and/or co-morbidities which could play a significant role in the emergence of mental distress and mental health issues in this population. Lastly, there may be a selection bias of men who choose surgery only—they may be more anxious (risk factor for depression), may not tolerate ambiguity, and may suffer more regret for making a hasty decision. This possibility provokes the question of what role personality may play in prostate cancer treatment decisions and risks for worse quality of life for the same functional outcomes [46].

Despite these limitations, however, the exploratory results reported here have merit and extend the existing literature. The insights gained from these data add a unique perspective to the existing body of literature about the contribution of cancer type, treatment modality, age, and multimorbidity to the presence or absence of depression or anxiety among cancer patients. While cross-sectional analyses are not ideal, given the paucity of prospective data evaluating potential factors that contribute to poor mental health among PCa patients, especially those treated with surgery [7], the data we present here sheds a light on the vulnerability of men with a history of prostate cancer. Further, the longitudinal nature of the Atlantic PATH and CanPath cohorts will allow for us to follow these prostate cancer survivors and their mental health and well-being in the long term. 

## 5. Conclusions 

The identification of factors that contribute to the mental distress of this population, especially among surgery patients, is a priority and can help tailor care and patient education and empowerment programs in urology to assist those most in need of support during the cancer survivorship journey [43]. Regular mental distress screening during survivorship, after radical prostatectomy, may be warranted.

## Figures and Tables

**Table 1:** Descriptive, logistic and correlational analyses predicting screening positive for depression or anxiety by type of history of cancer diagnosis, treatment modality, age, household income, current marital status, comorbidity and survivorship time among adult men residing in Atlantic Canada, aged 36+, between 2009 and 2015 (*n* = 281 screening positive for depression; *n* = 280 screening positive for anxiety).

	No Depression (*n* = 227, 84.7%) *n*, *%*	Mild, Moderate or Severe Depression (*n* = 41, 15.3%) *n*, *%*	No Anxiety (*n* = 227, 84.7%) *n*, *%*	Mild, Moderate or Severe Anxiety (*n* = 41, 15.3%) *n*, *%*
**History of Cancer Diagnosis**	*n* = 281, X^2^(1) = 5.17 *		*n* = 219, X^2^(1) = 1.36	
Prostate	46, *74.2*	16, *25.8*	53, *86.9*	8, *13.1*
Other	189, *86.3*	30, *13.7*	201, *91.8*	18, *8.2*
**Treatment Modality**	*n* = 264, X^2^(1) = 3.55		*n* = 263, X^2^(1) = 0.73	
Surgery	122, *87.7*	17, *12.2*	126, *92.0*	11, *8.0*
Other treatments (no surgery)	99, *79.2*	26, *20.8*	112, *88.9*	14, *11.1*
**Age**	*n* = 281, X^2^(1) = 2.22		*n* = 280, X^2^(1) = 5.18 *	
36–59 yrs old	85, *79.4*	22, *20.6*	89, *85.6*	15, *14.4*
60–69 yrs old	150, *86.2*	24, *13.8*	165, *93.8*	11, *6.2*
**Household Income**	*n* = 264, X^2^(2) = 11.61 **		*n* = 263, X^2^(2) = 14.06 **	
<$50,000	45, *69.2*	20, *30.8*	50, *79.4*	13, *20.6*
$50,000–99,999	98, *88.3*	13, *11.7*	104, *92.9*	8, *7.1*
$100,000+	76, *86.4*	12, *13.6*	85, *96.6*	3, *3.4*
**Current marital status**	*n* = 281, X^2^(1) = 17.84 ***		*n* = 280, X^2^(1) = 2.17	
Divorced, or single	17, *56.7*	13, *43.3*	25, *83.3*	5, *16.7*
Married or with partner	218, *86.9*	33, *13.1*	229, *91.6*	21, *8.4*
**Comorbidity**	*n* = 280, X^2^(2) = 11.29 **		*n* = 279, X^2^(2) = 10.38 **	
None	111, *89.5*	13, *10.5*	118, *95.2*	6, *4.8*
One	81, *84.4*	15, *15.6*	91, *91.0*	9, *9.3*
Two or more	42, *70.0*	18, *30.0*	44, *80.0*	11, *20.0*
**Survivorship time**	*n* = 281, r = 0.21		*n* = 280, r = −0.008	
Number of months since diagnosis; 0 to 69 months [*n*, M, (SD)]	235, *11.55 (14.25)*	46, *12.39 (16.95)*	254, *11.81 (14.57)*	26, *11.38 (17.78)*

Notes: *** *p* < 0.001; ** *p* < 0.01; * *p* < 0.05, two-tailed test.

**Table 2:** Stratified binary logistic regression analysis by treatment modality predicting screening positive for depression or anxiety by history of cancer and covariates among adult men residing in Atlantic Canada, aged 36+, between 2009 and 2015, (*n* = 286).

	***Surgery***	
	**Presence of Depression** ***n* = 171, OR (95% CI)/*[OR_MI_ (95% CI)]***	**Presence of Anxiety** ***n* = 170, OR (95% CI)/*[OR_MI_ (95% CI)]***
	X^2^(8) = 32.91 ***	X^2^(8) = 14.08
**History of Cancer Diagnosis**	X^2^(1) = 6.21 *	X^2^(1) = 0.02
Prostate	7.55 (1.54,37.04) */[5.09 (1.30,19.88)] *	0.85 (0.08, 9.05)/*[0.99 (0.10,10.37)]*
Other cancer types	1.0 Reference	1.0 Reference
**Age**	X^2^(1) = 0.13	X^2^(1) = 1.17
36–59 years old	1.30 (0.30,5.59)/*[1.97 (0.50,7.68)]*	2.45 (0.48,12.39)/*[2.68 (0.53,13.58)]*
60–69 years old	1.0 Reference	1.0 Reference
**Household Income**	X^2^(2) = 6.27 *	X^2^(2)= 5.57
<50K annually	5.57 (0.99,31.52)/*[2.75 (0.61,12.39)]*	12.06 (1.12,129.79) */*[10.04 (0.87,116.07)]*
50K to $99,999	0.86 (0.16,4.68)/*[0.83 (0.22,3.14)]*	2.73 (0.26,28.90)/*[2.66 (0.24,29.24)]*
100K+	1.0 Reference	1.0 Reference
**Current marital status**	X^2^(1) = 8.56 **	X^2^(1) = 0.16
Divorced, or single	16.48 (2.25,107.74) **/[6.18 (1.25,30.48)] *	1.54 (.19,12.26)/*[1.33 (0.17,10.75)]*
Married or with partner	1.0 Reference	1.0 Reference)
**Comorbidity**	X^2^(2) = 2.64	X^2^(2) = 2.00
One	2.67 (0.53,13.43)/*[1.65 (0.29,9.38)]*	0.88 (0.23,6.20)/*[0.72(0.11,4.75)]*
Two or more	3.94 (0.73,21.38)/*[4.08(0.98,17.01)]*	2.83 (0.52,15.46)/*[3.33 (0.67,16.48)]*
None	1.0 Reference	1.00 Reference
**Survivorship time**	X^2^(1) = 1.28	X^2^(1) = 0.62
Number of months since diagnosis	1.03 (0.98,1.07)/*[1.02 (0.99,1.05)]*	1.02 (0.98,1.06)/*[1.01 (0.97,1.05)]*
	***Other Forms of Active Cancer Treatment Modalities***	
	**Presence of Depression** ***n* = 115** **OR (95% CI)**	**Presence of Anxiety** ***n* = 116** **OR (95% CI)**
	X^2^(8) = 13.90	X^2^(8)= 26.52 **
**History of Cancer Diagnosis**	X^2^(1) = 1.09	X^2^(1)= 3.60
Prostate	1.83 (0.59,5.66)/[2.30(1.01,5.27)] *	5.18 (0.95,28.29)/*[6.24 (1.25,31.11)]* *
Other cancer types	1.0 Reference	1.0 Reference
**Age**	X^2^(1) = 2.17	X^2^(1) = 9.41 **
36–59 years old	2.11 (0.78,5.70)/*[1.91 (0.58,6.26)]*	16.31(2.74,97.08) **/*[13.42(2.56,70.28)]* **
60–69 years old	1.0 Reference	1.0 Reference
**Household Income**	X^2^(2) = 1.95	X^2^(2) = 4.19
<50K annually	0.88 (0.25,3.10)/*[1.22 (0.38,3.91)]*	8.31 (1.02,67.68) */*[7.83(0.98,62.68)]*
50K to $99,999	0.45 (0.13,1.55)/*[0.59 (0.18,1.95)]*	2.79 (0.33,23.42)/*[2.99 (0.35,25.32)]*
100K+	1.0 Reference	1.0 Reference
**Current marital status**	X^2^(1) = 3.02	X^2^(1) = 0.12
Divorced, or single	2.13 (0.86,11.43)/*[3.71 (1.04,13.28)]* *	1.37 (0.23,8.17)/*[0.89(0.17,4.78)]*
Married or with partner	1.0 Reference	1.0 Reference)
**Comorbidity**	X^2^(2) = 5.37	X^2^(2) = 7.98 *
One	1.46 (0.45,4.75)/*[1.59 (0.54,4.64)]*	8.69 (1.31,57.89) */*[5.82 (0.99,34.04)]*
Two or more	4.14 (1.23,14.00) **/*[4.28 (1.60,11.44)]* **	30.63(2.68,350.44) **/*[30.40(3.17,291.22)]* **
None	1.0 Reference	1.00 Reference
**Survivorship time**	X^2^(1) = 1.28	X^2^(1) = 0.22
Number of months since diagnosis	1.03 (0.98,1.07)/*[1.02 (0.97,1.07)]*	0.99 (0.93,1.05)/*[0.99(0.94,1.05)]*

Notes: *** *p* < 0.001; ** *p* < 0.01; * *p* < 0.05, two-tailed test.

## Data Availability

Data and biological samples from Atlantic PATH are available to researchers through a data access process.
